# Clinical outcome and intraprocedural characteristics of left atrial appendage occlusion: a comparison between single-occlusive plug-type and dual-occlusive disc-type devices

**DOI:** 10.3389/fcvm.2024.1401974

**Published:** 2024-07-18

**Authors:** Uwe Primessnig, Helene Schrader, Julia M. Wiedenhofer, Tobias D. Trippel, Abdul S. Parwani, Florian Blaschke, Gerhard Hindricks, Volkmar Falk, Henryk Dreger, Mohammad Sherif, Leif-Hendrik Boldt

**Affiliations:** ^1^ Deutsches Herzzentrum der Charité, Department of Cardiology, Angiology and Intensive Care Medicine, Campus Virchow Klinikum, Berlin, Germany; ^2^ Freie Universität Berlin and Humboldt-Universität zu Berlin, Charité—Universitätsmedizin Berlin, Berlin, Germany; ^3^ DZHK (German Centre for Cardiovascular Research), Berlin, Germany; ^4^ Department of Cardiology, Angiology and Intensive Care Medicine, Campus Charité Mitte, Deutsches Herzzentrum der Charité, Berlin, Germany; ^5^ Department of Cardiothoracic and Vascular Surgery, Deutsches Herzzentrum der Charité, Berlin, Germany

**Keywords:** left atrial appendage occlusion (LAAO), atrial fibrillation (AF), oral anticoagulation (OAC), thromboembolic prevention, transient ischemia attack (TIA), single-occlusive plug type (SOPT), dual-occlusive disc type (DODT)

## Abstract

**Background:**

Percutaneous interventional left atrial appendage occlusion (LAAO) is a reliable, safe, and effective alternative for stroke prevention in selected patients with atrial fibrillation (AF).

**Methods:**

In a retrospective observational study, 149 patients underwent LAAO between 2016 and 2022 at the Department of Cardiology of the Charité—Universitätsmedizin Berlin, Campus Virchow, with AF for prevention of thromboembolic complications. We compared patient characteristics, intraoperative details and postoperative outcomes between single-occlusive plug-type (SOPT) and dual-occlusive disc-type (DODT) devices.

**Results:**

In all patients, the device implantation was successful. 60 patients received a SOPT occluder, including Watchman (35%) and Watchman FLX Occluders (65%), while 89 patients received a DODT occluder, including Amplatzer Cardiac Plug (37.1%), the Amplatzer Amulet (25.8%), and the LAmbre occluder (37.1%) systems. Procedure duration was significantly longer for DODT occluder implantation (49 ± 33 vs. 41 ± 25 min, *p* = 0.018). There were no in-hospital deaths or thromboembolic events reported after LAAO in both groups. Beyond that, a low rate of bleeding or access-side-related complications and pericardial tamponades were observed. Anticoagulation at discharge varied. About 60.8% of patients received dual antiplatelet therapy at hospital discharge, and 33.1% received direct oral anticoagulants. A 6-month follow-up was obtained in 85% of the patients. All implanted devices were in the desired position. However, in 5.7% of the patients, a device-related thrombus formation was detected in the SOPT group, while no thrombus was seen in the DODT group (*p* = 0.11). Thromboembolic events were noticed in 3.1%, without any difference between the device types. There was a statistically non-significant trend for less residual device leaks after SOPT vs. DODT implantation (no leak in 71.7% vs. 62.2%, *p* = 0.07; minor leaks <5 mm, 9.4% vs. 20.3%, *p* = 0.1). In the SOPT group, less bleeding complications were reported after LAAO (11.3% vs. 17.6%, *p* = 0.1).

**Conclusion:**

Our data suggest the safety and efficiency of LAAO with a very high procedural implantation success rate irrespective of the used LAA device. Furthermore, no relevant procedural or device-related complication occurred during the 6-month follow-up in all patients.

## Introduction

Interventional percutaneous left atrial appendage occlusion (LAAO) represents a reasonable alternative to oral anticoagulation (OAC) for stroke prevention in selected patients with atrial fibrillation (AF) (1). Small randomized clinical trials showed the efficacy and safety of LAAO in AF patients with an increased risk for thromboembolic events (2–4). Non-inferiority was demonstrated, for example, in the Prague-17 randomised controlled trial (RCT) (5) trial comparing LAAO with standard oral anticoagulation therapy with new oral anticoagulants (NOACs) in the prevention of cardiovascular and neurological events. For AF patients who are not suitable for oral anticoagulation due to a history of major bleeding events or an increased risk of bleeding, percutaneous LAAO is an important possibility to reduce the risk of stroke and systemic thromboembolism (6–8). In a meta-analysis of the PREVAIL (3), the PROTECT AF (7), and the Prague-17 trial (5), a significant reduction in cardiovascular and all-cause deaths of LAAO when compared to OACs was observed (4). There are different endocardial LAAO devices available from various manufacturers. The main difference between the devices is their design. There are LAAO devices, which are designed as a single-occlusive plug-type (SOPT) device including the Watchman and the Watchman FLX. On the other hand, there are devices configured as dual-occlusive disc-type (DODT) systems, like the Amplatzer Cardiac Plug, the Amplatzer Amulet, which consist of a lobe and an additional disc, and the LAmbre system configured with an umbrella and a disc. All endocardial LAAO devices for percutaneous interventional implantation are available in different sizes (9).

In the present study, we aimed to evaluate the differences in two different occluder system designs in terms of intrahospital and 6-month clinical outcome, implantation success, and device-related complication rates. Therefore, we compared SOPT device, including the Watchman and the Watchman FLX occluder, vs. DODT systems, including the Amplatzer Cardiac Plug, the Amplatzer Amulet, and the LAmbre occluder systems.

## Methods

In this single-center, retrospective observational study,, we included 149 patients who underwent interventional percutaneous endocardial LAA occluder implantation between 2016 and 2022 at the Cardiology Department of the Charité—Universitätsmedizin Berlin, Campus Virchow, hospital. All patients had documented non-valvular, AF, a high thromboembolic risk [CHA2DS2-VASc ≥2)] with an indication for LAAO according to the current ESC guidelines (1, 10). All patients received a transesophageal echocardiography (TEE) before the LAAO procedure and up to 6 months after the procedure as a control examination for the correct LAA occluder device position and for exclusion of major leakage or thrombus formation. The procedure was performed according to current standards (11). The choice of the respective occluder device was left to the implanting physicians’ discretion.

The study was approved by the local ethical committee of the Charité (EA1/242/23). Clinical data were extracted retrospectively from electronic medical records. Complete case analysis was performed for all data points. All analyses were performed on pseudonymized datasets to protect patient privacy and confidentiality.

### Statistical analysis

Statistical analyses were performed using R 4.2.3 (R Core Team 2023). For continuous variables, normality was assessed using the Shapiro–Wilk test. Continuous variables are reported as means (± standard deviations) if normally distributed or as medians (interquartile ranges, IQR) if not normally distributed. Categorical variables are presented as absolute numbers and percentages. Student's *t*-test or Wilcoxon–Mann–Whitney *U*-test was employed for continuous variables, while the Chi-square test was used for categorical variables.

## Results

### Patient demographics

A total of 149 patients underwent percutaneous LAA occluder implantation between 2016 and 2022 at the Cardiology Department of the Charité—Universitätsmedizin Berlin, Campus Virchow, and were included in this analysis. Sixty of these patients received an SOPT occluder, including Watchman and Watchman FLX Occluders. Eighty-nine patients received a DODT occluder, including the Amplatzer Cardiac Plug, the Amplatzer Amulet and, the LAmbre occluder systems. Both groups showed balanced baseline characteristics, with a median age of 75.2 years and a proportion of females of 0.34. Cardiovascular and metabolic diseases including arterial hypertension, coronary artery disease, chronic kidney disease, diabetes, and obesity were equally distributed between both groups (Table 1). Atrial fibrillation was paroxysmal in 40.3% of patients, persistent in 44.3%, and permanent in 15.4%. About 95% of all patients received oral anticoagulation before implantation. In the group with SOPT occluder implantation, 81.7% of the patients had a history of bleeding, and in the group with DODT occluder implantation, it was 87.6%. LAAO was mainly triggered in both groups by gastrointestinal and intracranial bleedings followed by unstable international normalized ratio (INR) values. Other reasons for LAA occluder implantation were terminal kidney injury requiring dialysis; contraindications for oral anticoagulation other than previous bleeding, e.g., allergies, hemato-oncological conditions, recurrent stroke, or LAA thrombus despite oral anticoagulation; poor patient adherence toward medication; and, in two cases, patient request. All patients included in our study had a high risk for thromboembolic events and additionally suffered from a relevant risk of bleeding or some contraindications for long-term oral anticoagulation reflected in both groups as a mean CHA_2_DS_2_-VASc score of 5 and a mean HAS-BLED score of 3. All parameters are summarized in Table 1. More than half of the patients were in AF during LAAO. LVEF was normal to mildly reduced (53.0 ± 9.3%) and the left atrium (LA) was dilated with a mean LAVI of 45.8 ± 17.6 ml/m^2^. Most common LAA morphology was chicken wing anatomy in both groups (28.3% in the SOPT group and 46.1% in the DODT group). LAA size was slightly smaller in the DODT occluder group (19.1 ± 3.84 mm) than in the SOPT occluder group (20.6 ± 3.79 mm). Accordingly, the implanted device size was bigger in the SOPT group (25.8 ± 4.08 mm vs. 23.2 ± 4.66 mm, *p* < 0.001). Detailed characteristics are listed in Tables 2, 3.

**Table 1:** Baseline patient characteristics.

	Occluder systems		Total	*P*-value
	SOPT	DODT		
*N*	60	89	149	
Female	23 (38.3%)	28 (31.5%)	51 (34.2%)	0.49
Age (y)	74.5 (7.89)	75.7 (8.09)	75.2 (8.00)	0.46
BMI (kg/m²)	26.9 (4.93)	26.9 (4.99)	26.9 (4.95)	0.99
Arterial hypertension	53 (88.3%)	74 (83.1%)	127 (85.2%)	0.52
Coronary artery disease	32 (53.3%)	49 (55.1%)	81 (54.4%)	0.97
Peripheral arterial disease	9 (15.0%)	18 (20.2%)	27 (18.1%)	0.55
Hyperlipidemia	22 (36.7%)	40 (44.9%)	62 (41.6%)	0.40
Diabetes	23 (38.3%)	26 (29.2%)	49 (32.9%)	0.33
Obesity	19 (31.7%)	24 (27.0%)	43 (28.9%)	0.66
Chronic kidney disease	6 (10.0%)	13 (14.6%)	19 (12.8%)	0.56
Dialysis	5 (8.3%)	7 (7.9%)	12 (8.1%)	1
Liver disease	3 (21.4%)	7 (38.9%)	10 (31.3%)	0.50
Atrial fibrillation				
Paroxysmal	22 (36.7%)	38 (42.7%)	60 (40.3%)	0.57
Persistent	29 (48.3%)	37 (41.6%)	66 (44.3%)	0.52
Permanent	9 (15.0%)	14 (15.7%)	23 (15.4%)	1
CHA2DS2-VASc	5.00 [2.00–8.00]	5.00 [2.00–8.00]	5.00 [2.00–8.00]	0.60
HAS-BLED	3.00 [2.00–6.00]	3.00 [0–7.00]	3.00 [0–7.00]	0.80
History of bleeding	49 (81.7%)	78 (87.6%)	127 (85.2%)	0.44
Gastrointestinal bleeding	23 (46.9%)	46 (59.0%)	69 (54.3%)	0.25
Intracranial bleeding	12 (24.5%)	15 (19.2%)	27 (21.3%)	0.63
Unstable INR	9 (15.0%)	15 (16.9%)	24 (16.1%)	0.94
Poor mobility	5 (10.2%)	2 (2.6%)	7 (5.5%)	0.15
Other indications for LAAO	32 (58.2%)	46 (54.8%)	78 (56.1%)	0.82
Anticoagulation				
VKA	8 (14.0%)	9 (10.5%)	17 (11.9%)	0.70
NOAC	23 (40.4%)	44 (51.2%)	67 (46.9%)	0.27
Heparin (+derivatives)	5 (8.8%)	17 (19.8%)	22 (15.4%)	0.12
ASA ± P2Y12 antagonist	17 (29.8%)	19 (22.1%)	36 (25.2%)	0.40
No anticoagulation	3 (5.3%)	1 (1.2%)	4 (2.8%)	0.35

Values are displayed as frequencies (%), mean (standard deviation), or median [interquartile range].

VKA, vitamin K antagonists.

**Table 2:** Baseline rhythm and echocardiography parameters.

	Occluder system		Total	*P*-value
	SOPT	DODT		
*N*	60	89	149	
Rhythm				
SR	22 (40.0%)	28 (32.9%)	50 (35.7%)	0.50
AF	29 (52.7%)	47 (55.3%)	76 (54.3%)	0.90
Other atrial rhythm	2 (3.6%)	7 (8.2%)	9 (6.4%)	0.47
Cardiac pacing	2 (3.6%)	3 (3.5%)	5 (3.6%)	1
Heart rate	78.8 (22.0)	77.3 (25.4)	77.9 (24.1)	0.56
LVEF (%)	52.2 (10.1)	53.5 (8.77)	53.0 (9.33)	0.51
LAVI (ml/m²)	48.6 (17.6)	43.9 (17.5)	45.8 (17.6)	0.10
LAA size (mm)	20.6 (3.79)	19.1 (3.84)	19.7 (3.88)	0.03
LAA morphology				
Cactus	6 (10.0%)	8 (9.0%)	14 (9.4%)	1
Cauliflower	14 (23.3%)	18 (20.2%)	32 (21.5%)	0.80
Chickenwing	17 (28.3%)	41 (46.1%)	58 (38.9%)	0.04*
Doublewing	4 (6.7%)	6 (6.7%)	10 (6.7%)	1
Windsock	13 (21.7%)	11 (12.4%)	24 (16.1%)	0.20
Other	6 (10.0%)	5 (5.6%)	11 (7.4%)	0.49

Values are displayed as frequencies (percent) or mean (standard deviation).

SR, sinus rhythm.

*statistically significant.

**Table 3:** Procedural parameters.

	Occluder system		Total	*P*-value
	SOPT	DODT		
*N*	60	89	149	
Immediate success	60 (100%)	89 (100%)	149 (100%)	1
Procedural time (min)	40.9 (24.9)	49.1 (33.0)	45.7 (30.1)	0.018*
LAA device				
Amplatzer Cardiac Plug	0 (0%)	33 (37.1%)	33 (22.1%)	<0.001*
Amplatzer Amulet	0 (0%)	23 (25.8%)	23 (15.4%)	
LAmbre	0 (0%)	33 (37.1%)	33 (22.1%)	
Watchman	21 (35.0%)	0 (0%)	21 (14.1%)	
Watchman FLX	39 (65.0%)	0 (0%)	39 (26.2%)	
Device size (mm)	25.8 (4.08)	23.2 (4.66)	24.2 (4.60)	<0.001*
Contrast medium (ml)	69.4 (53.1)	66.8 (40.0)	67.8 (45.3)	0.99
Radiation time (s)	1,400 (1,830)	1,160 (1,280)	1,260 (1,520)	0.80
Device reposition ≥1	3 (5.0%)	9 (10.1%)	12 (8.1%)	0.41
Protrusion <10 mm	10 (16.7%)	1 (1.1%)	11 (7.4%)	0.001*

Values are displayed as frequencies (percent) or mean (standard deviation).

*statistically significant.

### Procedural parameters and clinical outcome

In all analyzed patients, successful occluder implantation was achieved. In the SOPT occluder group, 35% received a WATCHMAN Occluder and 65% received a WATCHMAN FLX Occluder. In the DODT occluder group, an Amplatzer Cardiac Plug was used in 37.1% of the cases, an Amplatzer Amulet in 25.8%, and a LifeTech LAmbre Occluder in 37.1%. Procedure time was significantly longer for DODT occluder implantation (49.1 ± 33.0 vs. 40.9 ± 24.9 min, *p* = 0.018). Three patients in the SOPT occluder group (5%) and nine patients (10.1%) in the DODT occluder group needed intraprocedural device reposition or change of the occluder type (*p* = 0.41). Device design–associated protrusion (<10 mm) into the LA was observed by transesophageal echocardiography at the end of the intervention and occurred more frequent in the SOPT occluder group including Watchman and Watchman FLX systems (16.7% vs. 1.1%, *p* = 0.001). No device-related thrombus was observed after the procedure. Furthermore, there were no significant differences in contrast medium quantity or radiation time. There were no in-hospital deaths or thromboembolic events reported after LAAO in both groups, beyond that a low rate of bleeding or access-side-related complications and pericardial tamponades were observed. Bleeding occurred in five patients in each group (8.3% in the SOPT group vs. 5.6% in the DODT group, *p* = 0.75), consisting of access-related and gastrointestinal bleedings. Pericardial tamponade, requiring percutaneous drainage, occurred in two patients, both in the DODT group (*p* = 0.16). Anticoagulation at discharge varied. Of the patients, 60.8% received dual antiplatelet therapy at hospital discharge and 33.1% received direct oral anticoagulants. More procedural details are listed in Tables 3, 4.

**Table 4:** Postprocedural outcome and in-hospital complications.

	Occluder system		Total	*P*-value
	SOPT	DODT		
*N*	60	89	149	
In-hospital death	0 (0%)	0 (0%)	0 (0%)	ns
Bleeding complication	5 (8.3%)	5 (5.6%)	10 (6.7%)	0.75
Device-related thrombus	0 (0%)	0 (0%)	0 (0%)	ns
Access-side complication	5 (8.3%)	6 (6.7%)	11 (7.4%)	0.96
TIA or stroke	0 (0%)	0 (0%)	0 (0%)	ns
Pericardial tamponade	0 (0%)	2 (2.2%)	2 (1.3%)	0.16
Postprocedural anticoagulation				
OAC (Vit-K AG)	2 (3.4%)	2 (2.2%)	4 (2.7%)	1
NOAC	17 (28.8%)	32 (36.0%)	49 (33.1%)	0.47
Dual antiplatelet (AS ± P2Y12AG)	40 (67.8%)	50 (56.2%)	90 (60.8%)	0.21
Heparin	1 (1.7%)	5 (9.0%)	6 (6.1%)	0.14

Values are displayed as frequencies (%) or mean (standard deviation).

### Short-term clinical outcome and mortality

A 6-month follow-up was obtained in 85% of the included patients (88% in SOPT and 83% in DODT). All implanted devices were in the desired position. However, in 5.7% of the patients, a device-related thrombus formation was detected in the SOPT group, while no thrombus was seen in the DODT group (*p* = 0.11). Thromboembolic events were noticed in 3.1%, without any difference between the device types. There was a trend for less residual device leaks after SOPT occluder implantation (no leak in 71.7% vs. 62.2%, *p* = 0.07). Minor leaks (<5 mm) were observed more often in the DODT group (20.3% vs. 9.4%, *p* = 0.1), without reaching statistical significance. In the SOPT group, less bleeding complications were reported after LAAO (11.3% vs. 17.6%, *p* = 0.1). Two patients died in the SOPT group and four died in the DODT group, reflecting an overall mortality of 4.7%. One death occurred due to intracranial bleeding under dual antiplatelet therapy, and the others were due to progression of preknown disease, including decompensated heart failure. Following this, to our knowledge, no death was directly related to the device. Details are summarized in Table 5.

**Table 5:** Short-term clinical outcomes (6 months).

	Occluder system		Total	*P*-value
	SOPT	DODT		
*N*	53	74	127	
Position				
Loco typico	53 (100%)	74 (100%)	127 (100%)	1
Leak				
None	38 (71.7%)	46 (62.2%)	84 (66.1%)	0.07
Minor (<5 mm)	5 (9.4%)	15 (20.3%)	20 (15.7%)	0.12
Major (≥5 mm)	0 (0%)	3 (4.1%)	3 (2.4%)	0.399
Device-related thrombus	3 (5.7%)	0 (0%)	3 (2.4%)	0.11
Bleeding events	6 (11.3%)	13 (17.6%)	19 (15.0%)	0.10
TIA or stroke	2 (3.8%)	2 (2.7%)	4 (3.1%)	1
Death	2 (3.8%)	4 (5.4%)	6 (4.7%)	0.414

Values are displayed as frequencies (%) or mean (standard deviation).

## Discussion

Elderly patients with different cardiovascular comorbidities suffering from paroxysmal, persistent, or permanent atrial fibrillation with high risk for thromboembolic events including stroke and transient ischemia attacks (TIAs) reflected with a mean CHA_2_DS_2_-VASc score of 4.7 ± 1.5 were included in this retrospective observational study. Furthermore, the patients’ study population—with a mean HAS-BLED score of 3.6 ± 1.2—were not suitable for long-term oral anticoagulation because of relevant bleeding complications, including mainly gastrointestinal, intracranial, or urogenital bleedings—followed by unstable INR values and contraindications for oral anticoagulation other than previous bleeding, e.g., hemato-oncological disease, recurrent stroke, or poor patient adherence toward medication and patient request. Our baseline data set reflects a patient population with a higher thromboembolic and bleeding risk compared to previous randomized clinical trials (2, 3). In the PROTECT AF trial, the mean CHA_2_DS_2_-VASc score was around 2.3, and in the PREVAIL trial, the mean CHA_2_DS_2_-VASc score ranged between 3.8 and 3.9. However, the EWOLUTION registry data reported that LAAO was safely performed in patients with a CHA_2_DS_2_-VASc score of 4.5 ± 1.6 (8).

Data from our present study suggest that percutaneous interventional left atrial appendage occlusion with either an SOPT, including the Watchman and Watchman FLX occluder, or DODT, including the Amplatzer Cardiac Plug, the Amplatzer Amulet, and the LAmbre occluder systems, is a safe and efficient alternative to oral anticoagulation independent of the used device configuration/design. To our knowledge, there are several studies that have compared different types of LAA occluders like Watchman, Amplatzer, or LAmbre devices (12), but this is the first study comparing SOPT and DODT occluder devices, irrespective of their manufacturer in terms of periprocedural performance, implantation success, postprocedural outcome, complication rate, and clinical follow-up.

Our real-world data demonstrate an overall procedural implantation success rate of 100% in our patients, which exceeds previous findings from the multicenter German LAARGE registry (13–15). This could be attributed not only to the high and long-term expertise of the operators in our department but also the availability of different occluder designs, so that our operators could choose the type of occluder that would—by their experience—best fit the individual patients LAA anatomy. However, this resulted in uneven distribution of the anatomical LAA variants between the groups, with a higher proportion of a chicken wing LAA morphology in the DODT device group compared to the SOPT device group (46.1% vs. 28.3%, *p* = 0.04). While there are no differences regarding patient characteristics, prevalence of AF, bleeding complications, anticoagulation regime prior to LAAO, and LAA morphology, the procedure duration was slightly longer in the DODT occluder group. Previous studies demonstrated either a longer procedure duration for the Amplatzer Amulet device compared to the Watchman device (16)—as in our study—or a similar procedure duration using Amplatzer Amulet and Watchman FLX devices (17). Contrast medium use was similar in our study between SOPT and DODT devices, while others reported a significantly lower use with the Amulet compared to the Watchman device (16). In contrast, Korsholm et al. showed lower contrast medium use in Watchman FLX compared to Amplatzer Amulet (17). In accordance with previous studies, fluoroscopy time did not differ in our study between both groups (13). While interpreting the results and findings from this retrospective analysis, it is important to acknowledge that in the SOPT group, 35% of the patients received a first-generation Watchman and 65% a Watchman FLX device. However, in the DODT occluder group, Amplatzer Cardiac Plug was used in 37.1% of the cases, Amplatzer Amulet in 25.8%, and LifeTech LAmbre occluder in 37.1%.

While no unsuccessful implantation occurred in our study, the LAA device had to be repositioned during the implantation in 8.1% of the procedures, with no difference between SOPT and DODT approaches. In the SOPT occluder group, the protrusion of the device into the LA occurred significantly more frequent compared to the DODT occluder group (16.7% vs. 1.1%, *p* = 0.001). In all our cases, the protrusion of the device into the LAA was less than 10 mm, and the devices did not interfere with the mitral valve or required any intervention. The safety of the LAAO with SOPT and DODT devices was confirmed in our cohort. We had no in-hospital deaths, strokes, or TIAs, and no device-related thrombus directly after procedure was reported. Bleeding complications were observed in 6.7% and access-side complications in 7.4%, irrespective of the device group. There were two pericardial tamponades in the DODT group, while none was reported in the SOPT group (1.3% with *p* = 0.16). Both patients were treated with sub-xiphoidal puncture, and no surgical intervention was necessary. Both groups of patients left the hospital without further sequelae. These findings are comparable to previous studies including the PREVAIL trial (3) where the rate of cardiac tamponades was reported to be 1.9%. Taken together, efficacy and safety were similar between the single-occlusive plug-type and the dual-occlusive disc-type systems, except for a higher percentage of non-relevant device protrusions in the LA in the SOPT group and is, therefore, in line with recently published prospective randomized Amulet IDE trial (18).

After LAAO, 60.8% of included patients received dual antiplatelet therapy for at least 3 months with no significant difference between SOPT and DODT patients at hospital discharge. Direct oral anticoagulation was given to 33.1% of the patients, irrespective of the device design, and the remaining patients were treated with vitamin K antagonists or heparin. These results are comparable with other randomized clinical trials (2, 3, 8) and the multicenter German LAARGE registry (13).

Six-month mid-term clinical outcome and mortality data were available for 85% of the patients included in our study. In all patients, the LAAO device, irrespective of the design, was in the designated position. Data suggest less residual device leaks after SOPT occluder implantation (no leak in 71.7% vs. 62.2%, *p* = 0.07). Minor leaks were observed in 20.3% in the DODT and in 9.4% in the SOPT group (*p* = 0.12). Major leaks (>5 mm) were reported in 4.1% of the patients (exclusively in the DODT group, *p* = 0.1). Cheung et al. reported about 27.5% of minor leaks after 45 days in their single-center retrospective analysis comparing different LAA occluder devices (Watchman, Amplatzer Cardiac Plug, Amplatzer Amulet, and LAmbre), while they observed 0.7% major significant peridevice leaks (12). Kretzler et al. showed in their registry that 1.4% in the Watchman group showed a residual peridevice jet >5 mm, and no residual jet was found in the Amulet group (16). Dukkipati et al. reported the relevance of minor leaks after LAAO for an increased risk of thromboembolism (19).

Thrombus formation on the device at follow-up could be detected via transesophageal echocardiography in 5.7% (*n* = 3) in the SOPT group, while no thrombus could be seen in the DODT group (*p* = 0.11). The ORIGINAL registry detected in 4.5% of Watchman patients a device-related thrombus compared to 0% in the Amulet group (16). In the ASA Plavix feasibility study (ASAP-Study) in 4%, treated with dual antiplatelet therapy after LAAO with the Watchman occluder, a device thrombus could be revealed (7). Chun et al. reported in a prospective single-center registry a low rate of device-associated thrombus formation with a dual antithrombotic treatment for only 6 weeks and subsequently switched to aspirin monotherapy (20). The EWOLUTION registry detected a device thrombus in 3.7% of all LAAO patients irrespective of the postprocedural antithrombotic regime (21). A large prospective registry demonstrated lower rates of bleeding complications after LAAO with aspirin monotherapy or even no therapy (22). In the randomized ASAP-TOO trial, Holmes et al. investigated single antiplatelet therapy or no therapy in LAAO vs. control patients (23).

There were no differences observed between the SOPT and the DODT groups in the occurrence of death, thromboembolic events including strokes and TIAs, or bleeding complications in the clinical follow-up of 6 months. Two patients died in the SOPT group and four in the DODT group; however, all deaths were not device associated. Numerically, but not statistically significant, there were fewer bleeding complications in the SOPT group (0.1% vs. 14.6%, *p* = 0.11), mainly driven by gastrointestinal bleeding.

Our findings from this retrospective analysis of 149 patients who underwent LAAO with either a single-occlusive plug-type device or a dual-occlusive disc-type device are consistent with the conclusions of previous randomized trials including the SWISS APERO study (24) and others (2, 3, 5) showing that both LAAO occlude designs are safe and effective and differences are only marginal and clinically not relevant. Thus, this study is an important scientific contribution from the randomized clinical trials, prospective registries, and retrospective studies, which could further increase the acceptance and of LAAO for thromboembolic stroke prevention in patients with atrial fibrillation, especially in subjects who are not suitable for systemic anticoagulation.

## Limitations

There are some acceptable but important limitations of our present study. The current study was designed as a single-center, retrospective observational study. Therefore, the data of this study rely on the analysis of existing medical records, introducing potential biases and limitations related to the collection and availability of data. Furthermore, the small sample size of the study without missing propensity matched analysis between the device groups is a relevant limitation.

Only 85% of the patients completed the 6-month follow-up including echocardiography. CT scans for position control were not obtained by default. Statistical power was negatively influenced by the small cohort regarding the conclusions about death, thromboembolic events, and bleeding complications. Moreover, the follow-up period of 6 months was too short to investigate long-term clinical outcomes of the LAAO patients sufficiently. Moreover, the heterogeneity of the postprocedural antithrombotic regimens makes the full assessment of LAAO benefit in this patient population difficult. Device choice was due to the operator’s discretion, which enables potential bias, especially since this led to an uneven distribution of the LAA anatomy types between the occluder groups. Different anatomical requirements during the implantation process and after could have influenced the outcome, especially when certain occluder types were used more often in challenging cases. Furthermore, the case number was too small to distinguish between different occluder generations, which could also potentially have an influence on the outcome.

## Conclusion

This retrospective observational study demonstrates that LAAO can be performed in everyday clinical routine with a very high procedural success rate and safety. Differences between both occluder designs are marginal and appear clinically not relevant.

## Data Availability

The original contributions presented in the study are included in the article/Supplementary Material, further inquiries can be directed to the corresponding author.
